# Structure of *Hordeum vulgare* NADPH-dependent thioredoxin reductase 2. Unwinding the reaction mechanism

**DOI:** 10.1107/S0907444909021817

**Published:** 2009-08-14

**Authors:** Kristine G. Kirkensgaard, Per Hägglund, Christine Finnie, Birte Svensson, Anette Henriksen

**Affiliations:** a Carlsberg Laboratory, Denmark; b Enzyme and Protein Chemistry, Department of Systems BioIogy, Technical University of Denmark, Denmark

**Keywords:** NADPH-dependent thioredoxin reductases, disulfide oxidoreductases, barley, germination, seed development, NADPH, redox regulation

## Abstract

Thioredoxins (Trxs) are protein disulfide reductases that regulate the intracellular redox environment and are important for seed germination in plants. Trxs are in turn regulated by NADPH-dependent thioredoxin reductases (NTRs), which provide reducing equivalents to Trx using NADPH to recycle Trxs to the active form. Here, the first crystal structure of a cereal NTR, *Hv*NTR2 from *Hordeum vulgare* (barley), is presented, which is also the first structure of a monocot plant NTR. The structure was determined at 2.6 Å resolution and refined to an *R*_cryst_ of 19.0% and an *R*_free_ of 23.8%. The dimeric protein is structurally similar to the structures of *At*NTR-B from *Arabidopsis thaliana* and other known low-molecular-weight NTRs. However, the relative position of the two NTR cofactor-binding domains, the FAD and the NADPH domains, is not the same. The NADPH domain is rotated by 25° and bent by a 38% closure relative to the FAD domain in comparison with *At*NTR-B. The structure may represent an intermediate between the two conformations described pre­viously: the flavin-oxidizing (FO) and the flavin-reducing (FR) conformations. Here, analysis of interdomain contacts as well as phylogenetic studies lead to the proposal of a new reaction scheme in which NTR–Trx interactions mediate the FO to FR transformation.

## Abstract 2

The first crystal structure of a cereal NTR, a protein involved in seed development and germination, has been determined. The structure is in a conformation that excludes NADPH binding and indicates that a domain reorientation facilitated by Trx binding precedes NADPH binding in the reaction mechanism.

## 1. Introduction

**Figure 1:**
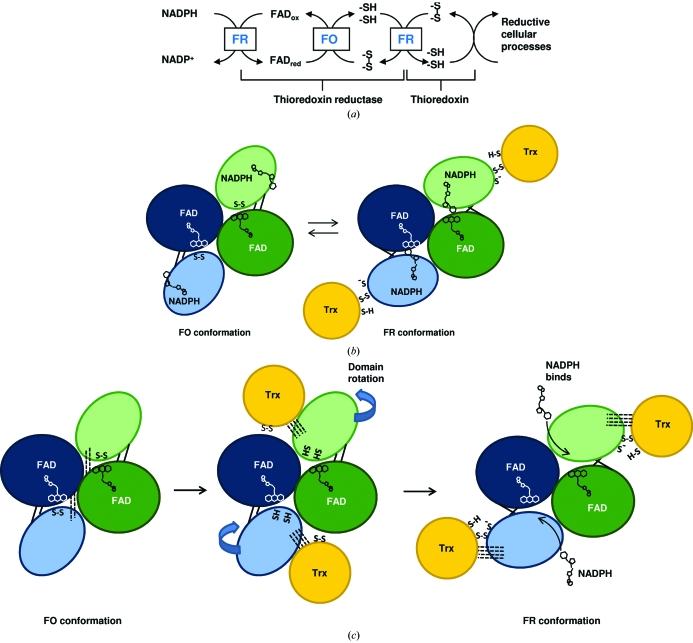
(*a*) Reaction catalyzed by NTR. Reducing equivalents are transferred from NADPH to FAD bound to NTR. From FAD they are transferred to a disulfide bond in the NADPH domain of NTR and further to a disulfide in the Trx substrate. In order to catalyze the entire reaction, NTR needs to swap between two conformations: the flavin-oxidizing (FO) and flavin-reducing (FR) conformations. The electron transfer linked to each conformation is framed. (*b*) A schematic view of the FO and FR conformations as proposed by Waksman *et al.* (1994 ▶). The two subunits in each NTR dimer are shown in blue and green, respectively. The darker coloured ovals symbolize the FAD domains, while the lighter coloured ovals show the NADPH domains. Disulfide and thiols are indicated as S-S and S-H, respectively. The black lines connecting the two domains symbolize the antiparallel β-sheets around which a 66° rotation occurs to bring NTR from the FO to the FR conformation. Thereby, the nicotine amide ring is positioned in proximity to the flavin isoalloxazine-ring system and the dithiols are brought to the surface of the protein where they can reduce Trx (shown in yellow). (*c*) The NTR reaction scheme modified to take the observation of differences in inter-domain interactions and lack of space for NADPH binding in the *Hv*NTR2 crystal structure into account. Hydrogen bonds are shown by dotted lines. Trx interaction is required for breakage of inter-domain contacts in the FO conformation and domain reorientation, and NADPH/NADP^+^ is assumed not to bind during domain reorientation.

Thioredoxin (Trx) systems are ubiquitous redox regulators that facilitate the reduction of other proteins *via* disulfide-exchange reactions (Fig. 1 ▶ *a*). In most organisms, Trx is reduced enzymatically by NADPH *via* NADPH-dependent thioredoxin reductase (NTR; Williams, 1976 ▶). The tripartite system of Trx, NTR and NADPH is known to be involved in DNA synthesis, oxidative-stress response and apoptosis (Arnér & Holmgren, 2000 ▶). Thus, reduced thioredoxin can activate ribo­nucleotide reductase (Laurent *et al.*, 1964 ▶; Moore *et al.*, 1964 ▶), methionine sulfoxide reductase (Russel & Model, 1986 ▶) and peroxi­redoxins (Tripathi *et al.*, 2009 ▶).

Plants exhibit a unique thioredoxin system with a complex time-, tissue- and organelle-specific expression pattern of a diverse selection of Trx isozymes that are not found in other organisms (Gelhaye *et al.*, 2004 ▶; Ishiwatari *et al.*, 1998 ▶). Furthermore, some plant Trxs are reduced by ferredoxin and ferredoxin reductase (FTR; de la Torre *et al.*, 1979 ▶) or by the glutaredoxin system: glutaredoxin (Grx), glutathione and glutathione reductase (GR; Gelhaye *et al.*, 2003 ▶). The NTR/Trx system in plants has a variety of functions and a wide range of target proteins have been identified by proteomics approaches (Hägglund *et al.*, 2008 ▶). Cytosolic Trx h plays important roles during seed germination by reducing disulfides in storage proteins and inhibitors of proteases and α-­amylases (Jiao *et al.*, 1993 ▶; Serrato & Cejudo, 2003 ▶; Wong *et al.*, 2004 ▶). Barley seeds contain two forms each of Trx h and NTR that have an overlapping spatio-temporal appearance and can interact interchangeably (Maeda *et al.*, 2003 ▶; Shahpiri *et al.*, 2008 ▶, 2009 ▶).

NTRs are members of the family of pyridine nucleotide-disulfide oxidoreductases (Pai, 1991 ▶) and contain two Rossmann-type domains that bind FAD and NADPH, respectively. NTRs from mammals and other higher eukaryotes are closely related to GR and are relatively rigid homodimeric proteins of >50 kDa. Each subunit contains three domains, of which the C-terminal domain forms the subunit interface (Manstein *et al.*, 1988 ▶; Waksman *et al.*, 1994 ▶; Williams *et al.*, 2000 ▶). Bacteria, yeast and plant NTRs (∼35 kDa) also contain two Rossmann-type nucleotide-binding domains, but they lack the extra C-­terminal domain. A subgroup of larger (51–58 kDa) chloro­plastidial NTRs contain an extra C-terminal domain with a Trx-like active-site motif CGPC (Alkhalfioui *et al.*, 2007 ▶; Serrato *et al.*, 2004 ▶). This domain is not related to the C-­terminal domain found in NTRs from higher eukaryotes and its presence defines the plant NTR-C subtype.

In the NTR-mediated reactivation of Trx, electrons are transferred from NADPH to Trx *via* a tightly bound FAD and a disulfide bridge (Mustacich & Powis, 2000 ▶). The active-site disulfide is found in the FAD domain in NTRs from higher eukaryotes and GRs and electron transfer occurs without any major structural changes. However, in the low-molecular-weight NTRs the disulfide is located in the NADPH domain and in the first crystal structure of the enzyme it is inaccessible to Trx in the so-called flavin-oxidizing conformation (FO), in which FAD is oriented for transfer of electrons to the NTR disulfide (Kuriyan *et al.*, 1991 ▶).

It was proposed that a 66° rotation about two β-strands connecting the FAD and the NADPH domains could expose the active-site cysteines and bring them into contact with the Trx active site and at the same time bring the FAD isoalloxazine into contact with NADPH for reduction (Waksman *et al.*, 1994 ▶; Fig. 1 ▶ *b*). The crystal structure of *Escherichia coli* NTR (*Ec*NTR) cross-linked to Trx demonstrated that the proposed reaction mechanism was indeed plausible (Lennon *et al.*, 2000 ▶). The complex illustrates how FAD is oriented for reduction by NADPH and the reduced active-site cysteines exposed for Trx binding in the so-called flavin-reducing (FR) conformation. In a previous study, Lennon and Williams showed that no single step in the reductive half-reaction of NTR was solely rate-limiting in turnover and reported a slight decrease in the observed rate constant for the rate-limiting step as a function of NADPH concentration. They proposed the FO to FR conformational change to be rate-limiting (Lennon & Williams, 1997 ▶).

Fifteen low-molecular-weight NTR structures have been deposited in the PDB; five of these are structures of *Ec*NTR (Kuriyan *et al.*, 1991 ▶; Lennon *et al.*, 1999 ▶, 2000 ▶; Waksman *et al.*, 1994 ▶). Eight other bacterial NTRs have had their structures determined (Akif *et al.*, 2005 ▶; Gustafsson *et al.*, 2007 ▶; Her­nandez *et al.*, 2008 ▶; Ruggiero *et al.*, 2009 ▶ and the unpublished deposition 2q7v; D. A. R. Sanders, J. Obiero, S. A. Bonderoff & M. M. Goertzen), while only one yeast (Zhang *et al.*, 2009 ▶) and one plant NTR, the *Arabidopsis thaliana* NTR-B (*At*NTR-B; Dai *et al.*, 1996 ▶), have been deposited. All deposited structures, except for the *Ec*NTR–Trx complex and the structure of *Thermoplasma acidophilum* NTR, which apparently does not need NADPH as an electron donor, show an NTR in the FO conformation.

The present analysis of the structural and functional properties of plant NTRs reports the structure of barley (*Hordeum vulgare*) NTR2 (*Hv*NTR2), the first structure of a monocot NTR, which moreover falls into a distinct phylogenetic class of NTRs (Shahpiri *et al.*, 2008 ▶). The overall structure of *Hv*NTR2 is found to be the same as previously reported for *Ec*NTR and *At*NTR-B, but has a different relative orientation of the FAD and NADPH domains which would interfere with NADPH binding as defined by the structure of *Ec*NTR with bound NADP^+^ or AADP^+^ (Lennon *et al.*, 2000 ▶; Waksman *et al.*, 1994 ▶). The results lead to the proposal that domain re­orientation facilitated by binding of Trx to the NTR FO state precedes the binding of NADPH.

## 2. Experimental procedures

### 2.1. Protein expression and purification

Recombinant *Hv*NTR2 was expressed in *E. coli* Rosetta (DE3) (Novagen) with an N-terminal His tag MGSSHHHH­HHSSGLVPRGSH as described previously (Shahpiri *et al.*, 2008 ▶). More specifically, His_6_-*Hv*NTR2 was purified on a HisTrap HP affinity column (GE Healthcare) pre-equilibrated with 10 m*M* imidazole, 0.5 *M* NaCl and 30 m*M* Tris–HCl pH 8.0 and eluted with a 0–100% gradient of 400 m*M* imidazole, 0.5 *M* NaCl and 30 m*M* Tris–HCl pH 8.0. Finally, the protein was dialyzed against 10 m*M* Tris–HCl pH 8.0, the purity was checked by SDS–PAGE and the sample was concentrated on an Amicon Ultra centrifugal filter unit (10 kDa molecular-weight cutoff; Millipore) to an OD_280_ of 3.96, which corresponds to a concentration of approximately 2.5 mg ml^−1^. The His_6_-*Hv*NTR2 solution was used for crystallization experiments without further purification and was not subjected to thrombin cleavage.

### 2.2. Crystallization and data collection

Initial crystallization screening experiments were carried out using the PEG 6000 Grid Screen (Hampton Research) and the hanging-drop vapour-diffusion method. Drops of 2.0 µl protein solution were mixed with 2.0 µl reservoir solution and equilibrated over a 500 µl reservoir. Yellow needles were detected in 5%(*w*/*v*) PEG 6000 (Fluka) and 0.1 *M* citrate buffer pH 4.0 after 4 d of incubation at 295 K. Fine-tuning of crystallization conditions included screening of the PEG concentration, the effect of the PEG molecular weight and use of the Hampton Research Additive Screen. The optimized conditions consisted of 24%(*w*/*v*) PEG 400, 2% Jeffamine M-­600, 0.1 *M* citrate buffer pH 3.5, a protein concentration of 5.7 mg ml^−1^ and an incubation temperature of 298 K. These conditions gave bright yellow crystals with hexagonal morph­ology within a week. The diameter of the crystals could reach 0.18 mm. The crystals were flash-frozen directly from the drop without using additional cryoprotectants.

The final X-ray data set was collected at 100 K on the ID14-­2 beamline at ESRF in Grenoble using a wavelength of 0.933 Å. A total of 120 frames were collected, each covering an oscillation width of 0.5°. The data were indexed and integrated using *MOSFLM* (Leslie, 1992 ▶) and scaled using the program *SCALA* from the *CCP*4 suite (Collaborative Computational Project, Number 4, 1994 ▶). The best crystal diffracted to a resolution of 2.6 Å and belonged to space group *P*6_2_22, with unit-cell parameters *a* = *b* = 133.7, *c* = 166.1 Å. Assuming the presence of two molecules in the asymmetric unit gave a Matthews coefficient of 2.90 Å^3^ Da^−1^ (Matthews, 1968 ▶). Final data-collection and processing statistics are summarized in Table 1 ▶.

### 2.3. Structure determination and refinement

Molecular replacement was performed with the program *MOLREP* (Vagin & Teplyakov, 2000 ▶) from *CCP*4 using the structure of *At*NTR-B as the initial search model. The *Hv*NTR2 and *At*NTR-B sequences are 75% identical. Significant molecular-replacement solutions were only found when the FAD and the NADPH domains were used as independent search models. The model was first refined using *REFMAC*5 (Murshudov *et al.*, 1997 ▶) and at later stages using *Phenix* (Adams *et al.*, 2002 ▶) and including TLS refinement interspaced with manual model rebuilding in *Coot* (Emsley & Cowtan, 2004 ▶) using the *Coot* validation procedures and *MolProbity* (Davis *et al.*, 2007 ▶) to correct problematic areas of the model. The final model had an *R*_cryst_ of 19.0% and an *R*_free_ of 23.8% based on 5% of the reflections. The *R*_free_ reflections were picked by random selection of reflections. The two molecules in the asymmetric unit, which do not represent the functional dimer, were divided into five TLS segments each using the *TLSMD* server (Painter & Merritt, 2006 ▶). The TLS segments in molecule *A* in the asymmetric unit are residues 6–71, 72–127, 128–181, 182–258 and 259–323. In molecule *B* the TLS segments cover residues 5–60, 61–127, 128–168, 169–255 and 256–323. The two first TLS segments in each molecule belong to the FAD domain, the following two belong to the NADPH domain and the last segment corresponds to the C-terminus of the FAD domain. Owing to the limited resolution of the data, only 48 solvent molecules were included and only where *F*_obs_ − *F*_calc_ electron density of  >3σ with optimal hydrogen-bonding distances to hydrogen donors or acceptors was found. Four citrate molecules were included in excess electron density owing to the appropriate size and geometry of this molecule and the presence of citrate in the crystallization conditions. Two citrate ions are bound in each NADPH domain. Some excess 2*F*_obs_ − *F*_calc_ electron density in the active site adjacent to the FAD isoalloxazine could not be satisfactorily modelled by solvent or citrate. Parameters for the refined model are summarized in Table 1 ▶. Solvent accessibility was calculated using *AREAIMOL* from the *CCP*4 suite with a 1.4 Å radius probe (Collaborative Computational Project, Number 4, 1994 ▶). Differences in domain orientation were analyzed using the *DynDom* server (Hayward & Lee, 2002 ▶). Superpositions were performed in *Coot* (Emsley & Cowtan, 2004 ▶). Inter-domain and ligand interactions were plotted using the program *LIGPLOT* (Wallace *et al.*, 1995 ▶). The molecular coordinates and structure factors have been deposited in the Protein Data Bank under the accession code 2whd.

## 3. Results and discussion

### 3.1. Structure quality

The final model of *Hv*NTR2 contains two molecules in the asymmetric unit, covering residues 6–323 (chain *A*) and 5–323 (chain *B*). The numbering refers to the amino-acid sequence of wild-type *Hv*NTR2. The biologically relevant dimer, inferred by analogy to the *E. coli* NTR system, is formed around the crystallographic twofold axis. The structure was determined at 2.6 Å resolution and refined to an *R*_cryst_ of 19.0% and an *R*_free_ of 23.8%. One FAD molecule with well defined electron density and *B* factors (∼40 Å^2^) comparable to the surrounding protein is present in each subunit. NADPH did not fit the excess electron density in the expected NADPH-binding pocket. Instead, the density fitted reasonably well to a citrate molecule accidentally present from the crystallization conditions (real-space *R* factor = 0.7–0.9). High *B* factors but continuous main-chain electron density is found in the N-terminus (residues 6–12), the loop between A1 and B3 (residues 33–35), B5 and surrounding loops (residues 96–105), the loop between B10 and B11 (residues 153–158), B12 and surrounding loops (residues 174–196) and B15 and surrounding loops (residues 220–245) (Supplementary Fig. 1^1^). The highest *B* factors were found in the C-terminal part of the FAD domain. The two molecules in the asymmetric unit can be superimposed with an r.m.s.d. of 0.1 Å for C^α^ atoms. The largest differences are found in the C-­terminal part of the FAD domain and especially in the loop between β-strands B18 and B19. Structure-quality parameters are summarized in Table 1 ▶.

### 3.2. Overall structure

**Figure 2:**
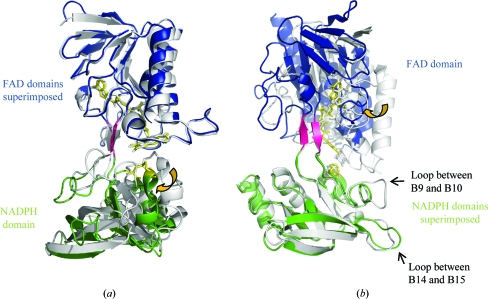
(*a*) Superposition of the FAD domains of *Hv*NTR2 (blue) and *At*NTR-B (white; PDB code 1vdc). The NADPH domains (green) were not included in the superposition. The *Hv*NTR2 FAD and the disulfide bridge are shown in yellow and the β-strand linker is shown in pink. (*b*) Superposition of the NADPH domains of *Hv*NTR2 (green) and *At*NTR-B (white; PDB code 1vdc). Yellow arrows indicate the relative change in domain arrangement between the two structures.

As in other low-molecular-weight NTRs, *Hv*NTR2 forms a homodimer with each subunit having two domains: the FAD and the NADPH domain. The two domains are quite similar, with 82 superimposable C^α^ atoms giving a root-mean-square deviation of 2.4 Å. The FAD domain consists of residues 1–­126 and 255–331 and has an α/β structure comprised of a central five-stranded parallel β-sheet flanked by a four-stranded β-sheet on one side and three α-helices on the other (Fig. 2 ▶ and Supplementary Fig. 2^1^). The NADPH domain consists of amino-acid residues 127–254; here, a similar five-stranded parallel β-sheet is flanked by a three-stranded β-­sheet on one side and two α-helices plus a third short α-­helix containing the active-site cysteines on the other side of the sheet. The two domains are connected by two antiparallel β-­strands (amino-acid residues 124–126 and 255–257), which as per tradition are assigned to the FAD domain (Fig. 2 ▶). Only a few inter-domain interactions stabilize the relative orientation of the two domains (see §[Sec sec3.7]3.7 and Table 2 ▶).

### 3.3. General NTR features

The overall structure of *Hv*NTR2 is the same as the structure of other low-molecular-weight NTRs. Superposition of *Hv*NTR2 C^α^ atoms with the structure of *At*NTR-B shows that that they are quite comparable, with root-mean-square deviations of 0.7 and 1.0 Å for the FAD and NADPH domains, respectively (calculated as least-square deviations using *Coot*). However, the relative orientation of the two domains in *Hv*NTR2 is quite different from their orientation in *At*NTR-B and other low-molecular-weight NTRs in the FO conformation (Fig. 2 ▶); the difference in orientation of the NADPH and FAD domains can be described by a 38.2% closure, a 1.0 Å translation and a 24.7° rotational twist. The rotation is centred about amino-acid residues 124–125 and 255–256, which are found in the short two-stranded β-sheet connecting the two domains, and shifts the orientation of the FAD molecule with respect to the active-site cysteines. The distance from Cys148 to the nearest reducing nitrogen in the iso­alloxazine rings is increased from the 3.4 Å observed in the structure of *At*NTR-B to 5.9 Å, the solvent accessibility of the FAD molecule is increased by 450% and that of the active-site disulfide is increased by 66%. The dimer assembly is not affected by the changed subunit conformation and FAD can still be reduced by NADPH as judged from the bleaching of the otherwise bright yellow colour of the crystals when they are subjected to a concentration of 10 m*M* NADPH for 30 min.

When the structure of *At*NTR-B is compared with that of the *Ec*NTR in the FR state (PDB code 1f6m), they differ by a minor translation of 1 Å and by a substantial 65.6° rotation about the two β-strands connecting the domains. However, comparing the structure of *Ec*NTR in the FR conformation with the structure of *Hv*NTR2 shows that they differ by a 6.7% closure, a translation of −1.4 Å and a rotation of 49.8°. The smaller rotation of 49.8° compared with 65.6° indicates that *Hv*NTR2 is actually closer to the FR conformation than other crystallized NTRs, which have all been in the FO conformation. Yet, within the group of NTR structures determined in the FO conformation there are minor variations in the relative orientation of the two domains. Superposition with *Ec*NTR requires an 8° inter-domain rotation for both *At*NTR-B and *Saccharomyces cerevisiae* NTR (*Sc*NTR; Zhang *et al.*, 2009 ▶) and an 11° rotation in the case of *Mycobacterium tuberculosis* NTR (*Mt*NTR; Akif *et al.*, 2005 ▶), indicating that the relative position of the two domains in the absence of a target sub­strate is quite flexible. A room-temperature structure of *At*NTR-B has been reported to be 2° off with respect to the relative orientation of the two domains compared with the deposited 98 K data (Dai *et al.*, 1996 ▶). Unfortunately, the coordinates from the room-temperature study have not been deposited in the PDB and it is not possible to relate this to the structural variation that we observe in *Hv*NTR2.

### 3.4. Plant-specific NTR motifs

**Figure 3:**
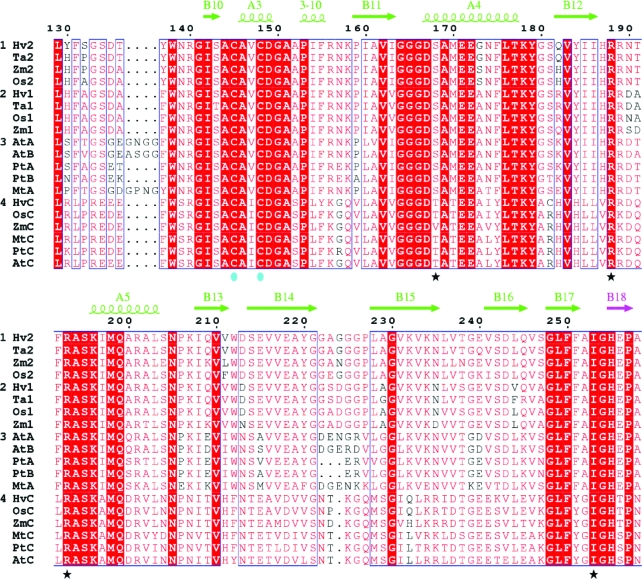
Segment of a sequence alignment of NTRs from different plants covering the two variable-loop segments in plant NTRs. The complete alignment can be found in Supplementary Fig. 1^1^. The NTRs, with their accession numbers given in parentheses, are *Hv*NTR1 (EU314717), *Hv*NTR2 (EU250021) and *Hv*NTRC from *Hordeum vulgare* (barley); *Ta*NTR1 (Q8VX47) and *Ta*NTR2 (TC297680) from *Triticum aestivum* (wheat); *Os*NTR1 (Q69PS6), *Os*NTR2 (Q6ZFU6) and *Os*NTRC (Q70G58) from *Oryza sativa* (rice); *Zm*NTR1 (EU966898), *Zm*NTR2 (BT054285) and *Zm*NTRC (BT037345) from *Zea mays* (maize); *At*NTRA (Q39242), *At*NTRB (Q39243) and *At*NTRC (O22229) from *Arabidopsis thaliana* (mouse-ear cress); *Pt*NTRA (AC149479), *Pt*NTRB (XM_002317595) and *Pt*NTRC (XM_002308899) from *Populus trichocarpa* (western balsam poplar); and *Mt*NTRA and *Mt*NTRC from *Medicago truncatula* (barrel medic, a legume). The sequences were aligned using *ClustalW* (Thompson *et al.*, 1994 ▶) and divided into four groups based on the phylogenetic analysis. Groups 1 and 2 are both monocotyledon subgroups of the A/B type, group 3 is the dicotyledon type A/B and group 4 is the subgroup of the C-type NTRs. Strictly conserved residues have a red background and residues that are well conserved within a group according to the Risler matrix (Risler *et al.*, 1988 ▶) are indicated by red letters. Residues conserved between groups are boxed. The secondary structure of *Hv*NTR2 was added using *ESPript* (Gouet *et al.*, 1999 ▶) and coloured according to domain: the NADPH domain is in green and the β-sheet linker between the two domains is in pink. Residues assumed to make hydrogen bonds to NADPH are marked by stars and the active-site cysteines are marked by cyan circles. The B9–B10 and the B14–B15 loops show the largest structural variation in a superposition of the *Hv*NTR2 and the *At*NTR-B structures. The primary structure of *At*NTR-B differs from the validated Q39243 sequence at positions 120 (I→T), 135 (V→A), 136 (L→S) and 329 (E→Q).

**Figure 4:**
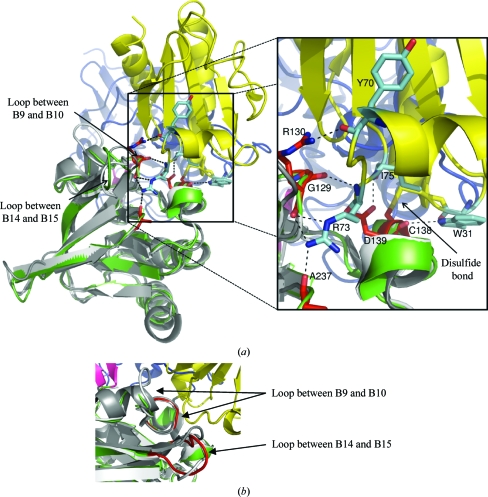
Superposition of the NADPH domains of *Hv*NTR2, *At*NTR-B (white; PDB code 1vdc) and *Ec*NTR in the FR conformation (grey; PDB code 1f6m) covalently bound to Trx (yellow). *Hv*NTR2 is coloured according to domain, with the FAD domain in blue, the NADPH domain in green and the β-sheet linker between the two domains in pink. (*a*) The hydrogen bonds between residues in *Ec*NTR (red) and Trx (cyan) are indicated by dotted lines. (*b*) Cartoon representation focused on the two loop areas with the largest structural variations. The loops of *Hv*NTR2 are coloured red.

The structure of *At*NTR-B is the only other plant NTR structure reported to date. As mentioned previously, the two proteins have 75% sequence identity. A superposition of the FAD domains (Fig. 2 ▶ *a*) shows a very similar orientation of loops, α-helices and β-sheets and the aforementioned variation in relative domain orientation. Some major structural differences are observed in two loop regions when the NADPH domains alone are superimposed (Fig. 2 ▶ *b*). The long loop region between strand B9 and B10 contains four additional residues in *At*NTR and therefore has a protrusion. This loop has the sequence S/N/P-F-T/V/A-G-S-G/E-E/K/T/D-G/A-N/P/S-G/N-G in dicot NTRs (the four extra residues are missing in *Populus trichocarpa*), while monocot NTRs of the A/B type have a H/Y-F-S/P/A-G-S-D-T/A sequence (Fig. 3 ▶ and Supplementary Fig. 2^1^). The second variable loop is located between β-strands B14 and B15. This loop is glycine-rich in *Hv*NTR2 and other monocot NTRs, in which a G-G-A/E/S-N/G/D-G-G-P-L-A/G motif is found. The corresponding loop in dicots appears to be variable in sequence and length. Both loops are expected to face the incoming Trx substrate molecule (Fig. 4 ▶).

The sequence combination in the two loops appears to vary between isoforms from the same species and the combined effect of the variation in the loops might result in the observed species-dependent interaction between NTR and Trx (Jacquot *et al.*, 1994 ▶; Rivera-Madrid *et al.*, 1995 ▶; Shahpiri *et al.*, 2008 ▶) and could indicate that Trx sub­strate specificity could be somewhat differentiated *via* these loops. All monocots included in the phylogenetic analysis of the plant NTRs have two low-molecular-weight NTRs of the A/B-type clustering in different sub­groups (Fig. 3 ▶ and Supplementary Fig. 2^1^). In con­trast, dicot NTRs of the A/B type appear to be more similar and are not subdivided. Both monocots and dicots express a single NTR of the C type, which has been characterized as chloroplast-specific (Al­khalfioui *et al.*, 2007 ▶; Serrato *et al.*, 2004 ▶).

### 3.5. FAD binding

The FAD-binding domain encloses the FAD between its two nonsequential halves, with the FMN part buried in the first half of the domain. Both hydrogen bonds (eight amino-acid residues contributing ten hydrogen bonds; Ser18, Ala21, Ile27, Gln52, Asn61, Val94, Asp293 and Ala302) and van der Waals interactions (involving 25 amino-acid residues) contribute to FAD binding. The hydrogen-bonding residues are conserved among the plant NTRs but are not conserved among all NTRs (Supplementary Fig. 2^1^). Only a few conservative substitutions are found among the van der Waals interacting residues, *e.g.* *At*NTR-B residues Val14 and Ile120 are substituted by *Hv*NTR2 residues Ile16 and Thr122, respectively.

### 3.6. NADPH binding

The binding of NADP^+^ to *Ec*NTR in the FO conformation (PDB code 1tdf) was used for comparison with the potential NADPH-binding pockets of *At*NTR-B and *Hv*NTR2. The residues involved in the binding of NADP^+^ in *Ec*NTR and potentially in *Hv*NTR2 and *At*NTR-B are listed in Supplementary Table 1^1^. All of the likely NADPH-binding residues are identical in *Hv*NTR2 and *At*NTR-B and only a few con­servative substitutions are found when compared with the actual binding pocket of *Ec*NTR.

A sulfate ion was found in the NADPH-binding pocket in the *At*NTR-B crystal structure and the partly occupied NADPH molecule also found in the pocket was in a distorted NADPH-binding mode. The likely binding of a citrate ion in the *Hv*NTR2 NADPH-binding pocket not only occludes NADPH binding but could also be the cause of the observed change in the relative domain orientation, which obstructs any possibility of NADPH binding owing to spatial limitations. Also, the unassigned electron density below the iso­alloxazine-ring system in the *Hv*NTR2 structure might influence the twist and closure in the domain structure.

The overall charge distributions and shapes of the *Ec*NTR and *Hv*NTR2 NADPH-binding pockets were examined and showed very similar charge distributions, with a large number of positive charges matching the negative charges of the NADPH phosphates. However, the superposition also showed that there is not enough space in the *Hv*NTR2 NADPH pocket to accommodate the ribose moiety of NADP^+^ owing to the changed orientation of the FAD domain. Thus, if *Hv*NTR2 represents an intermediate between the FO and the FR states, NADPH would have to undergo a considerable conformational change during catalysis. It appears likely that NADPH binds following the conformational change, which would also be in agreement with the pre­viously observed slight decrease in the observed rate constant for the domain reorientation event with increasing NADPH concentration (Lennon & Williams, 1997 ▶). The NADPH-binding pocket is fully solvent-accessible in the FR conformation (PDB code 1f6m). However, it is also possible that the observed domain orientation only reflects the binding of citrate in the active site and therefore is of no relevance to the reaction mechanism.

### 3.7. Inter-domain contacts

**Figure 5:**
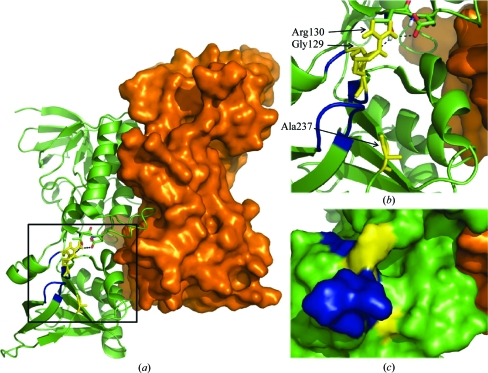
Thioredoxin-binding patch defined by the covalent *Ec*NTR–Trx complex. (*a*) The *Ec*NTR dimer in the FO conformation (PDB code 1tde). One subunit is shown in green and the solvent-accessible surface of the other is shown in orange. Residues Gly129 and Arg130 in the NADPH domain form the only hydrogen bonds to the FAD domain in the FO conformation. These residues (yellow) together with Ala237 provide all five of the hydrogen bonds formed upon Trx binding to the FR conformation. The residues interacting in the dimer interface are adjacent to the two loops (blue) that possibly provide some selectivity towards Trx isoforms. The association of Trx with this area prior to the conformational shift would break the inter-domain hydrogen bonds and thereby facilitate the shift. (*b*) A close-up view with hydrogen bonds indicated as dotted lines. (*c*) Solvent-accessible surface of the same area.

The inter-domain contacts in the FO conformation were mapped for *Ec*NTR (PDB code 1tde), *At*NTR-B and *Hv*NTR2 (Table 2 ▶). The hydrogen bonds between the two domains in *Ec*NTR originate from the loop between strands B9 and B10. Here, Gly129 and Arg130 form bonds to Thr47 and Glu48, respectively, in the FAD domain (Table 2 ▶ and Fig. 5 ▶). A third hydrogen bond connects Gln42 from the FAD domain to Ala116 in the hinge region. The inter-domain contacts are dis­located by two residues in *At*NTR-B, but involve the same loop. Here, Trp140 and Asn141 from the NADPH domain form hydrogen bonds to Thr53 in the FAD domain. As in *Ec*NTR, a third hydrogen bond between Gln50 and Ala124 connects the FAD domain to the hinge region. The residues involved in hydrogen bonds between domains are conserved in the *Hv*NTR2 and *At*NTR-B primary sequences, but since the domains are in different relative orientations the same hydrogen bonds cannot be formed. Only two inter-domain hydrogen bonds are found in *Hv*NTR2, both of which are mediated through the hinge region.

Nonbonded (van der Waals) interactions are located in the Gly129 and Thr47 area in *Ec*NTR and there are additional interactions between residue Phe142 in α-helix A3 carrying the active cysteines and Glu50 in the FAD domain. In *At*NTR-B the only inter-domain van der Waals interaction is between Glu258 in the hinge region and Lys125 located very nearby in the NADPH domain; similarly, in *Hv*NTR2 the van der Waals interactions are mediated through the hinge region only. These are from Glu256 to Arg127 of the NADPH domain, from His255 to Arg300 of the FAD-binding domain and from Val125 to Asn45 and Ile47 of the FAD-binding domain.

### 3.8. The reaction mechanism

The main inter-domain contacts in the FO conformation in *Ec*NTR and *At*NTR-B are centred on the loop between β-­strands B9 and B10. This loop contains an arginine residue (Arg130 in *Ec*NTR) that is conserved in plant NTR sequences (Arg142 in *At*NTR-B and Arg140 in *Hv*NTR2; Fig. 3 ▶). It is also found in most NTR sequences from other species, but can be substituted by lysine or asparagine. Arg130 forms three of the seven hydrogen bonds to Trx upon binding of the substrate in the *Ec*NTR FR conformation (PDB code 1f6m). The neighbouring Gly129 and Ala237 within its spatial proximity are each involved in one hydrogen bond to Trx. The last two hydrogen bonds engage the active-site amino-acid residues Cys138 and Asp139 (Fig. 4 ▶).

This patch, which adjoins the variable loops in the NADPH domain, supplies all hydrogen bonds specific for Trx binding besides those in the active site. The same area provides the interactions for anchoring of the NADPH domain to the FAD domain in the FO state in both *Ec*NTR and *At*NTR-B. If Trx binds to this patch in the FO conformation, the main anchoring between the domains will be broken and thereby two hydrogen bonds are replaced by four to five new ones in the NTR–Trx interface (Fig. 1 ▶ *c*). The binding of Trx could be guided by the two variable loops, ensuring binding to the optimal Trx isoform. The loop area is free to interact with Trx as observed for the FO conformation of *Ec*NTR (Fig. 5 ▶).

A third loop found between strand B3 and a short 3_10_-helix has been predicted to bind to Trx (Zhang *et al.*, 2009 ▶). Dicot NTRs have a strictly conserved E-G-W-M-A-N-D-I-A-P-G-G sequence in this area, while monocot NTRs display a greater sequence variation and invariably have the proline exchanged for an alanine. The C-type plant NTRs have a loop which is one amino-acid residue shorter in this region and has the consensus motif E-G-Y/C-Q-M/V-G-G-V-P-G-G. Simultaneous binding of Trx to this loop and active-site cysteines would require the NTR domain twist to have occurred.

Association of Trx with the FO conformation prior to NADPH binding might help in defining the NADPH-binding site. Our postulation that Trx breaks the inter-domain contacts as the first part of the reaction mechanism implies that the NTR domain rotation only happens, or only happens sufficiently, when Trx is available and would explain why almost all NTRs crystallized to date have been in the FO conformation. If the structure of *Hv*NTR2 is an intermediate between the FO and the FR conformations, it shows that there is not room for bound NADPH during the domain-rotation step. The con­formational change from the FO to the FR state could be part of the mechanism that secures the release of NADP^+^ from FR.

## Supplementary Material

PDB reference: *Hv*NTR2, 2whd, r2whdsf

Supplementary material file. DOI: 10.1107/S0907444909021817/be5129sup1.pdf

## Figures and Tables

**Table 1:** Data-collection and refinement statistics Values in parentheses are for the highest resolution bin.

Data collection	
Resolution (Å)	49.9–2.60 (2.74–2.60)
No. of unique reflections	27423 (3937)
Redundancy	7.1 (7.3)
Completeness (%)	99.6 (99.6)
*R*_merge_† (%)	6.6 (35.0)
〈*I*/σ(*I*)〉	18.4 (4.4)
Wilson *B* factor (Å^2^)	59.8
Refinement	
No. of amino-acid residues	635
No. of water molecules	48
*R*_cryst_‡ (%)	19.0
*R*_free_‡ (%)	23.8
Estimated coordinate error (Å)	0.33
R.m.s.d. from ideal geometry	
Bonds (Å)	0.011
Angles (°)	1.329
*B* factors (Å^2^)	
Protein (chain *A*/chain *B*)	59.2/60.9
FAD	39.3
Solvent	43.8
Ramachandran plot	
Most favoured (%)	82.8
Additionally allowed (%)	16.1
Generously allowed (%)	1.1
Disallowed (%)	0.0

† *R*_merge_ = 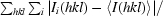 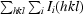, where 〈*I*(*hkl*)〉 is the mean intensity of *i* reflections with intensity *I*_*i*_(*hkl*).

‡ *R*_cryst_ = 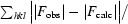 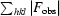, where *F*_obs_ and *F*_calc_ are observed and calculated structure factors, respectively. For *R*_free_, the sum is extended over a subset of reflections (5%) that were excluded from all stages of refinement.

**Table 2:** Inter-domain contacts in the FO states of *Ec*NTR (PDB code 1tde, no NADPH bound), *At*NTR-B and *Hv*NTR2

	NADPH domain	FAD domain	Hinge region	Distance (Å)	Interaction type
*Ec*NTR	Gly129	Thr47		3.1	Hydrogen bond
	Arg130	Glu48		3.0	Hydrogen bond
		Gln42	Ala116	3.1	Hydrogen bond
	Gly129	Thr47		3.9	van der Waals
	Phe142	Glu50		3.3/3.9/3.8	van der Waals
*At*NTR-B	Trp140	Thr53		2.7	Hydrogen bond
	Asn141	Thr53		3.1	Hydrogen bond
		Gln50	Ala124	2.9	Hydrogen bond
	Lys125		Glu258	3.4	van der Waals
*Hv*NTR2		Tyr273	His255	3.1	Hydrogen bond
	Arg127		Glu256	3.3	Hydrogen bond
	Arg127		Glu256	3.8	van der Waals
		Arg300	His255	3.7/3.7	van der Waals
		Asn45	Val125	3.8	van der Waals
		Ile47	Val125	3.8	van der Waals

## Footnotes

1 Supplementary material has been deposited in the IUCr electronic archive (Reference: BE5129). Services for accessing this material are described at the back of the journal.
