# Supplementary material for: Structure of Hordeum vulgare NADPH-dependent thioredoxin reductase 2. Unwinding the reaction mechanism
Source: Acta Crystallogr D Biol Crystallogr. 2009 Aug 14;65(Pt 9):932–41. doi: 10.1107/S0907444909021817 (PMC2733882; doi:10.1107/S0907444909021817)
Supplement: Supplementary file 1 [file d-65-00932-sup1.pdf]

Supplementary material

Figure 1. The *Hv*NTR2 SigmaA weighted 2Fo-Fc electron density contoured at a 1σ level and covering the (A) B9-B10 loop and the (B) B14-B15 loop, respectively.

Figure 2. Sequence alignment of NTRs from different plants. The NTRs and their accession numbers in parentheses are *Hv*NTR1 (EU314717), *Hv*NTR2 (EU250021) and *Hv*NTRC from *Hordeum vulgare* (barley), *Ta*NTR1 (Q8VX47) and *Ta*NTR2 (TC297680) from *Triticum aestivum* (wheat), *Os*NTR1 (Q69PS6), *Os*NTR2 (Q6ZFU6) and *Os*NTRC (Q70G58) from *Oryza sativa* (rice), *Zm*NTR1 (EU966898), *Zm*NTR2 (BT054285) and *Zm*NTRC (BT037345) from *Zea mays* (maize), *At*NTRA (Q39242), *At*NTRB (Q39243) and *At*NTRC (O22229) from *Arabidopsis thaliana* (mouse-ear cress), *Pt*NTRA (AC149479), *Pt*NTRB (XM_002317595) and *Pt*NTRC (XM_002308899) from *Populus trichocarpa* (western balsam poplar) and *Mt*NTRA and *Mt*NTRC from *Medicago truncatula* (Barrel Medic, legume). The sequences where aligned using ClustalW and divided into 5 groups. Group 1 and 2 are both monocotyledon subgroups of the A/B type, group 3 is the dicotyledons type A/B, and group 4 and 5 are the monocotyledon and dicotyledon subgroups of the C type, respectively. Residues strictly conserved have a red background, residues well conserved within a group according to the Risler matrix are indicated by red letters, residues conserved between groups are boxed and residues conserved within a group, but showing significant differences between groups, have an orange background. The secondary structure of *Hv*NTR2 was added using ESPript (Gouet *et al.*, 1999), and coloured according to domain; blue is the FAD domain, green the NADPH domain and pink are the β-sheets functioning as a linker between the two. Residues making hydrogen bond to FAD are indicated by triangles, residues assumed to make hydrogen bonds to NADPH by stars and the active site cysteines by cyan circles. The alignment is followed by the phylogentic tree produced by the same ClustalW analysis and illustrated in TreeView.

Figure 1

A

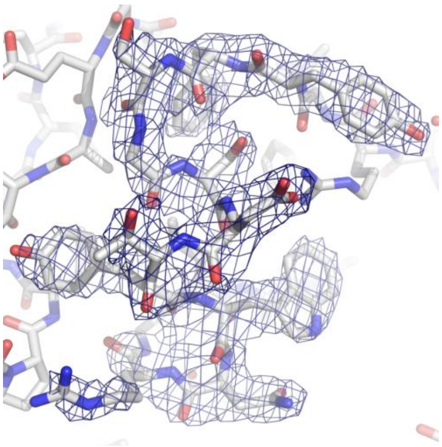

B

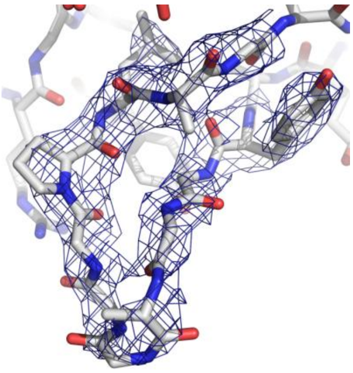

Figure 2

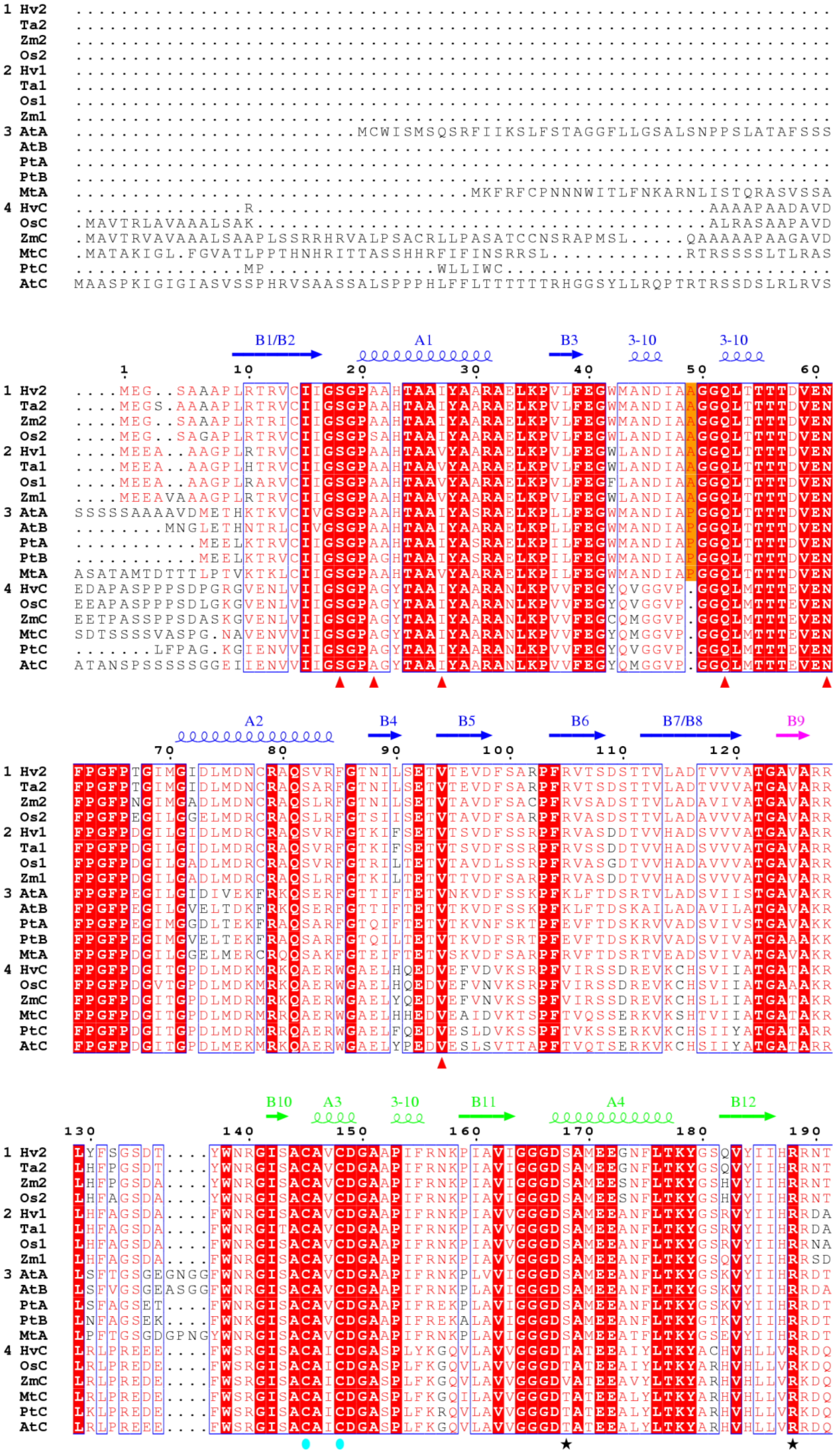

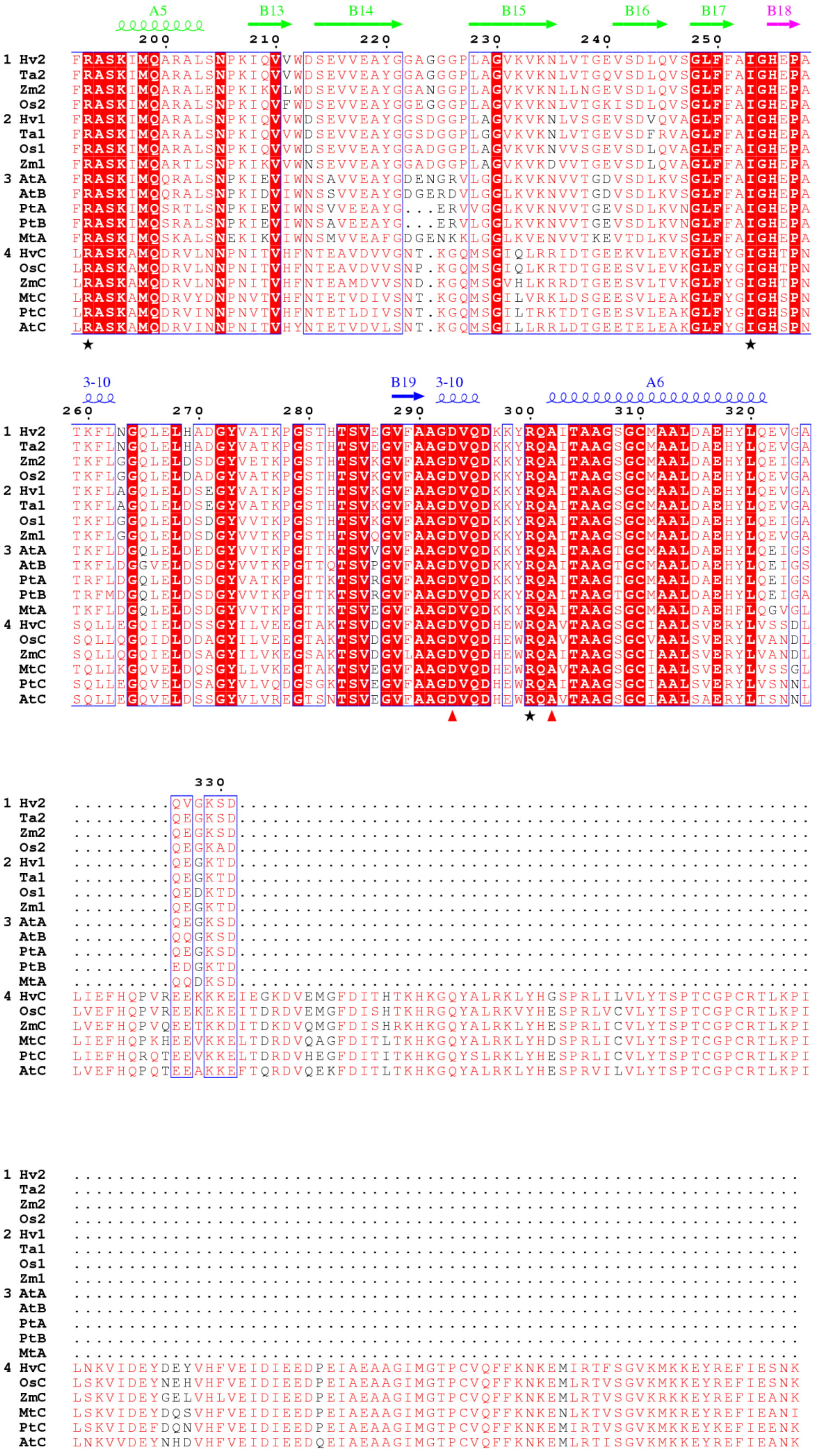
A5 B13 B14 B15 B16 B17 B18
200 210 220 230 240 250
1 Hv2 FRASKIMQARALSNPKIQVVWDSEVVEAYGGAGGGPLAGVKVKNLVTGEVSDLQVSGLFFAIGHEPA
Ta2 FRASKIMQARALSNPKIQVVWDSEVVEAYGGAGGGPLAGVKVKNLVTGQVSDLQVSGLFFAIGHEPA
Zm2 FRASKIMQARALENPKIKVLWDSEVVEAYGGANGGPLAGVKVKNLLNGEVSDLQVSGLFFAIGHEPA
Os2 FRASKIMQARALSNPKIQVFWDSEVVEAYGGEGGGPLAGVKVKNLVTGKISDLQVSGLFFAIGHEPA
2 Hv1 FRASKIMQARALSNPKIQVVWDSEVVEAYGGSDGGPLAGVKVKNLVSGEVSDVQVAGLFFAIGHEPA
Ta1 FRASKIMQARALSNPKIQVVWDSEVVEAYGGSDGGPLGGVKVKNLVTGEVSDFRVAGLFFAIGHEPA
Os1 FRASKIMQARALSNPKIQVVWDSEVVEAYGGADGGPLAGVKVKNVVSGEVSDLQVAGLFFAIGHEPA
Zm1 FRASKIMQARTLSNPKIKVVWNSEVVEAYGGADGGPLAGVKVKDVVTGEVSDLQVAGLFFAIGHEPA
3 AtA FRASKIMQQRALSNPKIEVIWNSAVVEAYGDENGRVLGGLKVKNVVTGDVSDLKVSGLFFAIGHEPA
AtB FRASKIMQQRALSNPKIDVIWNSSVVEAYGDGERDVLGGLKVKNVVTGDVSDLKVSGLFFAIGHEPA
PtA FRASKIMQSRTLSNPKIEVIWNSVVEEAYG...ERVVGGLKVKNVVTGEVSDLKVNGLFFAIGHEPA
PtB FRASKIMQSRALSNPKIEVIWNSAVEEAYG...ERVLGGLKVKNVVTGEVSDLKVNGLFFAIGHEPA
MtA FRASKIMQSKALSNEKIKVIWNSMVVEAFGDGENKKLGGLKVENVVTKEVTDLKVSGLFFAIGHEPA
4 HvC LRASKAMQDRVLNNPNITVHFNTEAVDVVGNT.KGQMSGIQLRRIDTGEEKVLEVKGLFYGIGHTPN
OsC LRASKAMQDRVLNNPNITVHFNTEAVDVVSNP.KGQMSGIQLKRTDTGEESVLEVKGLFYGIGHTPN
ZmC LRASKAMQDRVLNNPNITVHFNTEAMDVVSND.KGQMSGVHLKRRDTGEESVLTVKGLFYGIGHTPN
MtC LRASKAMQDRVYDNPNVTVHFNTETVDIVSNT.KGQMSGILVRKLDSGEESVLEAKGLFYGIGHSPN
PtC LRASKAMQDRVINNPNVTVHFNTETLDIVSNT.KGQMSGILTRKTDTGEESVLEAKGLFYGIGHSPN
AtC LRASKAMQDRVINNPNITVHYNTETVDVLSNT.KGQMSGILLRRLDTGEETELEAKGLFYGIGHSPN
★ ★
3-10 B19 3-10 A6
260 270 280 290 300 310 320
1 Hv2 TKFLNGQLELHADGYVATKPGSTHTSVEGVFAAGDVQDKKYRQAITAAGSGCMAALDAEHYLQEVGA
Ta2 TKFLNGQLELHADGYVATKPGSTHTSVEGVFAAGDVQDKKYRQAITAAGSGCMAALDAEHYLQEVGA
Zm2 TKFLGGQLELDSDGYVETKPGSTHTSVKGVFAAGDVQDKKYRQAITAAGSGCMAALDAEHYLQEIGA
Os2 TKFLGGQLELDADGYVATKPGSTHTSVKGVFAAGDVQDKKYRQAITAAGSGCMAALDAEHYLQEVGA
2 Hv1 TKFLAGQLELDSEGYVATKPGSTHTSVKGVFAAGDVQDKKYRQAITAAGSGCMAALDAEHYLQEVGA
Ta1 TKFLAGQLELDSEGYVATKPGSTHTSVKGVFAAGDVQDKKYRQAITAAGSGCMAALDAEHYLQEVGA
Os1 TKFLGGQLELDSDGYVVTKPGSTHTSVKGVFAAGDVQDKKYRQAITAAGSGCMAALDAEHYLQEIGA
Zm1 TKFLGGQLELDSDGYVVTKPGSTHTSVQGVFAAGDVQDKKYRQAITAAGSGCMAALDAEHYLQEVGA
3 AtA TKFLDGQLELDEDGYVVTKPGTTKTSVVGVFAAGDVQDKKYRQAITAAGTGCMAALDAEHYLQEIGS
AtB TKFLDGGVELDSDGYVVTKPGTTQTSVPGVFAAGDVQDKKYRQAITAAGTGCMAALDAEHYLQEIGS
PtA TRFLDGQLELDSDGYVATKPGTTKTSVRGVFAAGDVQDKKYRQAITAAGTGCMAALDAEHYLQEIGS
PtB TRFMDGQLELDSDGYVVTKPGTTKTSVRGVFAAGDVQDKKYRQAITAAGTGCMAALDAEHYLQEIGA
MtA TKFLDGQLELDSDGYVVTKPGTTKTSVEGVFAAGDVQDKKYRQAITAAGSGCMAALDAEHFLQGVGL
4 HvC SQLLEGQIELDSSGYILVEEGTAKTSVDGVFAAGDVQDHEWRQAVTAAGSGCIAALSVERYLVSSDL
OsC SQLLQGQIDLDDAGYILVEEGTAKTSVDGVFAAGDVQDHEWRQAVTAAGSGCVAALSVERYLVANDL
ZmC SQLLQGQIELDSAGYILVKEGSAKTSVDGVLAAGDVQDHEWRQAITAAGSGCIAALSVERYLVANDL
MtC TQLLKGQVELDQSGYLLVKEGTAKTSVEGVFAAGDVQDHEWRQAVTAAGSGCIAALSVERYLVSSGL
PtC SQLLEGQVELDSAGYVLVQDGSGKTSVEGVFAAGDVQDHEWRQAITAAGSGCIAALSVERYLVSNNL
AtC SQLLEGQVELDSSGYVLVREGTSNTSVEGVFAAGDVQDHEWRQAVTAAGSGCIAALSAERYLTSNNL
★
330
1 Hv2 ....QVGKSD..
Ta2 ....QEGKSD..
Zm2 ....QEGKSD..
Os2 ....QEGKAD..
2 Hv1 ....QEGKTD..
Ta1 ....QEGKTD..
Os1 ....QEDKTD..
Zm1 ....QEGKTD..
3 AtA ....QEGKSD..
AtB ....QQGKSD..
PtA ....QEGKSD..
PtB ....EDGKTD..
MtA ....QQDKSD..
4 HvC LIEFHQPVREEKKKEIEGKDVEMGFDITHTKHKGQYALRKLYHGSPRLILVLYTSPTCGPCRTLKPI
OsC LVEFHQPVREEKEKEITDRDVEMGFDISHTKHRGQYALRKVYHESPRLVCVLYTSPTCGPCRTLKPI
ZmC LVEFHQPVQEETKKDITDKDVQMGFDISHRKHKGQYALRKLYHESPRLICVLYTSPTCGPCRTLKPI
MtC LIEFHQPKHEEVKKELTDRDVQAGFDITLTKHKGQYALRKLYHDSPRLICVLYTSPTCGPCRTLKPI
PtC LIEFHQRQTEEVKKELTDRDVHEGFDITITKHKGQYSLRKLYHESPRLICVLYTSPTCGPCRTLKPI
AtC LVEFHQPQTEEAKKEFTQRDVQEKFDITLTKHKGQYALRKLYHESPRVILVLYTSPTCGPCRTLKPI
1 Hv2 ....
Ta2 ....
Zm2 ....
Os2 ....
2 Hv1 ....
Ta1 ....
Os1 ....
Zm1 ....
3 AtA ....
AtB ....
PtA ....
PtB ....
MtA ....
4 HvC LNKVIDEYDEYVHFVEIDIEEDPEIAEAAGIMGTPCVQFFKNKEMIRTFSGVKMKKEYREFIESNK
OsC LSKVIDEYNEHVHFVEIDIEEDPEIAEAAGIMGTPCVQFFKNKEMLRTVSGVKMKKEYREFIESNK
ZmC LSKVIDEYGELVHLVEIDIEEDPEIAEAAGIMGTPCVQFFKNKEMLRTVSGVKRKKEYREFIEANK
MtC LSKVIDEYDQSVHFVEIDIEEDPEIAEAAGIMGTPCVQFFKNKENLKTVSGVKMKREYREFIEANI
PtC LSKVIDEFDQNVHFVEIDIEEDPEIAEAAGIMGTPCVQFFKNKEMIRTVSGVKMKKEYKEFIEENK
AtC LNKVVDEYNHDVHFVEIDIEEDQEIAEAAGIMGTPCVQFFKNKEMLRTISGVKMKKEYREFIEANK

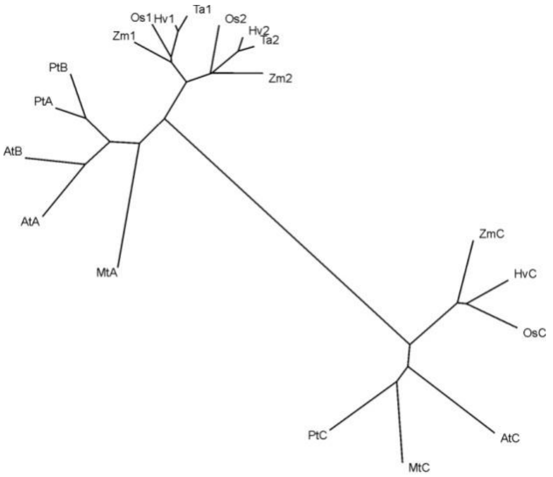

Os1
Hv1
Ta1
Os2
Hv2
Ta2
Zm1
Zm2
PtB
PtA
AtB
AtA
MtA
ZmC
HvC
OsC
PtC
MtC
AtC

Table 1 Amino acid residues involved in NADP+ binding to *Ec*NTR in the FO conformation (pdb accession: 1tdf) and the corresponding residues of *Hv*NTR2 and *At*NTR-B identified by superposition of the three structures (Wallace *et al.*, 1995).

*Ec*NTR	*Hv*NTR2	*At*NTR-B	Interaction type
Thr156	Ser168	Ser170	Two hydrogen bonds
Arg176	Arg188	Arg190	Two hydrogen bonds
Arg181	Arg193	Arg195	Hydrogen bond
Ile243	Ile253	Ile255	Hydrogen bond
Arg293	Arg300	Arg302	Hydrogen bond
Leu119	Leu129	Leu127	van der Waals
Ile151	Ile163	Ile165	van der Waals
Gly154	Gly166	Gly168	van der Waals
Glu159	Glu171	Glu173	van der Waals
His175	His187	His189	van der Waals
Arg177	Arg189	Arg191	van der Waals
Glu183	Ser195	Ser197	van der Waals
Gly244	Gly254	Gly256	van der Waals
His245	His255	His257	van der Waals
Asn260	*Asp271*	*Asp273*	van der Waals
Tyr262	*Tyr273*	*Tyr275*	van der Waals
His290	*Lys297*	*Lys299*	van der Waals

Hydrogen bond cut-off < 3.35Å and hydrophobic interaction cut-off < 3.90 Å. Residues in italics belong to the FAD domain.
